# Reference intervals for thyroid stimulating hormone and free thyroxine derived from neonates undergoing routine screening for congenital hypothyroidism at a university teaching hospital in Nairobi, Kenya: a cross sectional study

**DOI:** 10.1186/s12902-016-0107-9

**Published:** 2016-05-23

**Authors:** Geoffrey Omuse, Ali Kassim, Francis Kiigu, Syeda Ra’ana Hussain, Mary Limbe

**Affiliations:** Department of Pathology, Aga Khan University Hospital, P.O. Box 30270-00100, Nairobi, Kenya; Department of Paediatrics, Aga Khan University Hospital, P.O. Box 30270-00100, Nairobi, Kenya

**Keywords:** Neonatal reference intervals, free thyroxine (fT4), Thyroid stimulating hormone (TSH), Congenital hypothyroidism (CH)

## Abstract

**Background:**

In order to accurately interpret neonatal thyroid function tests (TFTs), it is necessary to have population specific reference intervals (RIs) as there is significant variation across different populations possibly due to genetic, environmental or analytical issues. Despite the importance of RIs, globally there are very few publications on RIs for neonatal TFTs primarily due to ethical and technical issues surrounding recruitment of neonates for a prospective study. To the best of our knowledge, this is the first report from Africa on neonatal RIs for TFTs.

**Methods:**

We used hospital based data largely derived from neonates attending the wellness clinic at the Aga Khan University Hospital Nairobi (AKUHN) where screening for congenital hypothyroidism is routinely done. Specifically we derived age and gender stratified RIs for free thyroxine (fT4) and thyroid stimulating hormone (TSH) which had been analyzed on a Roche e601 analyzer from 2011 to 2013. Determination of reference intervals was done using a non-parametric method.

**Results:**

A total of 1639 and 1329 non duplicate TSH and fT4 values respectively were used to derive RIs. There was a decline in TSH and fT4 levels with increase in age. Compared to the Roche RIs, the derived RIs for TSH in neonates aged 0–6 days and those aged 7–30 days had lower upper limits and narrower RIs. The fT4 lower limits for neonates less than 7 days and those aged 7–30 days were higher than those proposed by Roche. There was a significant difference in TSH RIs between male and female neonates aged less than 15 days. No gender differences were seen for all other age stratifications for both TSH and fT4. Appropriate age and gender specific RIs were subsequently determined.

**Conclusion:**

The AKUHN derived RIs for fT4 and TSH revealed similar age related trends to what has been published. However, the differences seen in upper and lower limits across different age stratifications when compared to the Roche RIs highlight the need for population specific RIs for TFTs especially when setting up a screening programme for congenital hypothyroidism. We subsequently recommend the adoption of the derived RIs by the AKUHN laboratory and hope that the RIs obtained can serve as a reference for the African population.

## Background

Several pediatric reference intervals (RIs) for thyroid function tests (TFTs) have been published [1–7]. The neonatal RIs described in some of these studies were established on the basis of relatively small numbers of subjects or using analyzers with diverse measurement principles and analytical performance. For example, in earlier studies ultrasensitive immunoassays were not used to measure thyroid stimulating hormone (TSH) [3]. The use of TSH immunoassays that can accurately determine very low concentrations has resulted in a change in published RIs and resulted in the introduction of diagnoses such as subclinical hyperthyroidism. Use of TSH assays with different test methodologies has been shown to influence reported incidence of congenital hypothyroidism (CH) [8]. Despite the importance of screening for CH in the neonatal period, there are very few studies that specifically address RIs in this age group.

CH is one of the most common preventable causes of mental retardation. Its overall incidence ranges from 1 in 3000 to 1 in 4000 newborn infants [9, 10]. The most common cause of CH is a primary disorder of the thyroid gland where reduced function from dysgenesis or dyshormonogenesis results in an increase in TSH concentration [11]. TFTs play an important role in screening for CH before the onset of symptoms hence enabling the institution of early treatment which has been associated with better clinical outcomes [12]. The prevalence of CH has been reported to have increased after the introduction of screening tests [13–15]. Several factors could contribute to a variation in reported prevalence of CH across different populations. Some of these factors include race and ethnicity [15]. Furthermore, the testing algorithm adopted when screening for CH as well as the RIs used can contribute to the variation in CH prevalence. Initially, many screening programs performed a thyroxine (T4) test, with a follow-up TSH test on infants with values below a specified T4 cutoff. This strategy not only identified primary CH but was able to identify neonates with secondary hypothyroidism. However, with advancements in the sensitivity of TSH assays, there has been a move towards the use of TSH as a screening test [12]. This is because serum TSH has a log-linear relationship with circulating thyroid hormone levels with a 2-fold change in free thyroxine (fT4) producing a 100-fold change in TSH [16]. Some programs have undertaken pilot programs measuring both fT4 and TSH on all newborns resulting in a higher diagnostic rate of congenital hypothyroidism [17].

It is recommended by the international federation of clinical chemistry (IFCC) that RIs should be population-specific and derived from a set of reference individuals representative of a reference population [18]. The clinical laboratory standards institute (CLSI) also recommends derivation of RIs through a formal study where samples are collected from a reference group comprising a minimum of 120 individuals identified from a reference population through probability sampling and use of non-parametric statistical methods to derive RIs. It also recommends transference or verification of RIs as an option in the event that establishing population specific RIs is not possible [19]. For a CH screening program, the laboratory carrying out testing should use TFT cut offs derived from the local population to ensure that they are appropriate since misdiagnosis can easily result from the adoption of inappropriate RIs.

At the Aga Khan University Hospital Nairobi (AKUHN), TFTs are performed on the Roche e601 analyzer. The cut offs recommended by Roche for fT4 and TSH in babies less than 3 months of age were derived from only 223 and 222 babies respectively from Leipzig, Germany [20]. This population was comprised of primarily a Caucasian population which was quite different from the AKUHN population which is largely comprised of black Africans.

We therefore set out to derive age and gender specific neonatal RIs for fT4 and TSH at AKUHN and compare them with the manufacturer’s intervals to determine whether any differences existed.

## Methods

### Study site

The study was carried out at AKUHN which is a Joint Commission International accredited (JCIA) 300 bed private hospital. It is a not-for-profit institution that provides both primary and tertiary health care services. It has state of the art intensive care and high dependency units for children and adults as well as a laboratory that has attained International Organization for Standardization (ISO) 15189:2007 accreditation since July 2011. The hospital also runs a number of general and specialized outpatient clinics. Among these is the Well Baby Clinic where infants are followed up. Since newborn screening is not yet a policy in Kenya, the hospital routinely encourages parents to have their babies screened for CH and have blood samples drawn on the fourth or fifth day of life, though some babies get screened later than this, but still within the newborn period, for different reasons. For pre-terms, routine screening for CH is only performed on attainment of an age equivalent to a term baby. Data on TSH and fT4 for neonates was obtained from the hospital health management system from February 2011 to December 2013. Specifically, we extracted consecutive data for all neonates who had fT4 or TSH done during the specified time period. Most of the neonatal TFTs are done as part of routine screening for CH and it was thought that this would serve as an ideal reference population assuming that neonates attending a wellness clinic will most likely be healthy. A neonate was defined as any newborn 30 days of age and below. This was used as a criterion when extracting data from the hospital health management system. File reviews were carried out only for neonates with TSH or fT4 outside the Roche RIs so as to identify and exclude from statistical analysis those with a diagnosis of a thyroid disorder or an acute illness. Neonates with both fT4 and TSH values outside Roche RIs were excluded from the study.

### Ethical approval

Informed consent from the patients whose laboratory data was used was not required as this study was classified as a clinical audit according to the hospitals research guidelines. Ethical waiver was obtained from the AKUHNs health research ethics committee (2013/REC-15).

### Thyroid function test determination

TFTs were carried out on the Roche e601 analyzer (Roche diagnostic GmbH, Mannheim, Germany) which uses the electro-chemiluminescence immunoassay principle to determine the concentrations of fT4 and TSH. Samples used were serum or plasma. The e601 TSH assay is a third generation immunoassay according to the definition by Spencer et al. [21]. Its functional sensitivity is 0.014 μIU/mL and limit of detection 0.005 μIU/mL. The coefficient of variation (CV) for fT4 was 1.97 % and 2.98 % at concentrations of 15.4 pmol/L and 55.3 pmol/L respectively. For TSH, the CVs were 2.64 % and 2.42 % at concentrations of 3.4 μIU/mL and 13.6 μIU/mL respectively. Both the assays are enrolled for the Randox International Quality Assessment Scheme (RIQAS) and performance has been satisfactory over the period for which the data was obtained.

### Data analysis

Reference interval determination was performed using Reference Value Advisor v2.1 (National Veterinary School, Toulouse, France) [22]. This is a free set of Excel macros that compute reference intervals from data contained in a spreadsheet. It carries out both parametric and non-parametric analysis for all data sets provided. The non-parametric derived RIs that capture the mid 95 % of reference values were used to determine RIs for this study. Initially, the data was stratified into 2 age groups 0–6 days and 7–30 days to enable comparison with Roche RIs. Subsequently, further age and gender stratification was done and comparisons using Mann–Whitney *U* test or Kruskal-Wallis H test were performed to determine differences between groups. Median or mean rank values were compared as appropriate. The age-wise stratification for determining RIs was 0–7 days, 8–14 days, 15–22 days and 23–30 days. Comparisons that showed no statistically significant difference after the independent samples median test were grouped together. Inferential statistical analysis was performed using IBM International Business Machines Statistical Package for the Social Sciences (IBM SPSS) Statistics for Windows, Version 21.0. (Armonk, NewYork, IBM Corporation). For all RIs, 90 % confidence limits were calculated for both the lower (2.5^th^ percentile) and upper limits (97.5^th^ percentile). RIs were also determined using standard and robust parametric methods to compare with the non-parametrically derived RIs. This was done on both untransformed data and data transformed using the Box-Cox method. Testing for normality was done using Anderson-Darling and a test for symmetry was performed for the robust method. Outliers and suspect data were determined using the Tukey’s method depending on whether values were less or greater than 3 times the inter-quartile range (IQR) or 1.5–3 times the IQR relative to the first and third quartiles. The Clinical Laboratory Standards Institute (CLSI) guidelines for determining reference intervals were used for this analysis [19]. P-values less than 0.05 were considered significant.

## Results

A total of 1673 and 1359 non duplicate values for TSH and fT4 respectively were obtained, however 34 TSH and 30 fT4 values respectively were excluded after review of 119 medical records and all TFT results. A total of 29 patients had both fT4 and TSH values excluded. The main reasons for exclusion included presence of an acute illness such as neonatal sepsis or jaundice, both fT4 and TSH values outside the Roche RIs and discrepancies in age. Subsequently, 1639 TSH and 1329 fT4 values were used in determining RIs.

**Fig. 1:**
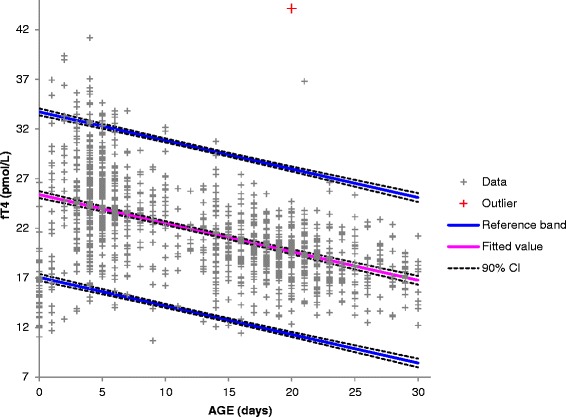
Distribution of TSH values in relation to age

There was a general decline in TSH with increase in age as shown in Fig. 1. The proposed Roche RIs for neonates in the age group 0–6 days is 0.7–15.2 μIU/ml and 0.72–11.0 μIU/ml for the age group 7–30 days. Out of 632 neonates in the age group 0–6 days, 38 (6.0 %) fell outside the Roche RIs, with 9 (1.4 %) having values above the upper limit. In the age group 7 to 30 days, out of 1009 neonates, 15 (1.5 %) fell outside the Roche reference limits with only 1 (0.1 %) having a value above the upper limit.

**Fig. 2:**
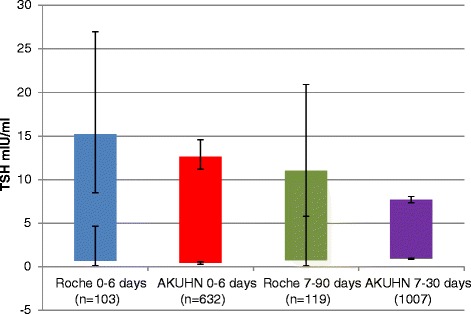
Roche and AKUHN neonatal TSH reference intervals with 90 % confidence limits

Compared to the Roche RIs, the derived RI for TSH in the 0–6 day age group was lower with narrower confidence limits around the upper and lower limits as shown in Fig. 2. For the 7–30 day old neonates, the derived RI had a narrower spread and lower upper limit with narrower confidence limits around the upper and lower limits as shown in Fig. 2. Age stratified comparison of medians showed no statistically significant difference between neonates aged 0–7 days and 8–14 days as well as those aged 15–22 days and 23–30 days. These were subsequently grouped into 0–14 days and 15–30 days respectively for further analysis. There was a statistically significant difference in TSH mean ranks between neonates aged 0–14 days and those aged 15–30 days (*U* = 312129, *p* = .029). There was a statistically significant difference in TSH mean ranks between male and female neonates aged 0–14 days (*U* = 87542, *p* = .002). Age and gender stratified TSH RIs are shown in Table 1.

**Table 1:** AKUHN non-parametrically derived neonatal reference intervals for thyroid stimulating hormone (TSH)

Age (days)	Number(gender)	Lower Limit (μIU/mL) (90 % confidence limits)	Median	Upper Limit (μIU/mL) (90 % confidence limits)
0 to 14	415 (M)	0.59 (0.42–0.77)	3.31	12.84 (10.62–14.44)
0 to 14	479 (F)	0.56 (0.42–0.69)	2.74	11.00 (9.56–12.33)
15 to 30	359 (M)	0.90 (0.83–0.97-0.94)	2.71	7.46 (7.01–7.83)
	386 (F)			

Key: *M* Male, *F* Female

The TSH RIs derived parametrically after data transformation and exclusion of outliers were similar to the non-parametrically derived RIs. However, most of the data failed to normalize after Box-Cox transformation hence the non-parametrically derived RIs were deemed most appropriate.

For fT4, the suggested Roche RIs are 11.0–32.0 pmol/L and 11.5–28.3 pmol/L for neonates in the age groups 0–6 days and 7–30 days respectively. Out of 515 neonates in the age group 0–6 days, 43 (8.3 %) had values outside the Roche RIs with all of them being above the upper limit. In neonates aged 7–30 days, 6 out of 814 (0.7 %) had fT4 values outside the Roche RIs with 2 (0.6 %) having values below the lower limit. Four of the 5 had corresponding TSH results with only 1 being above the Roche upper limit.

**Fig. 3:**
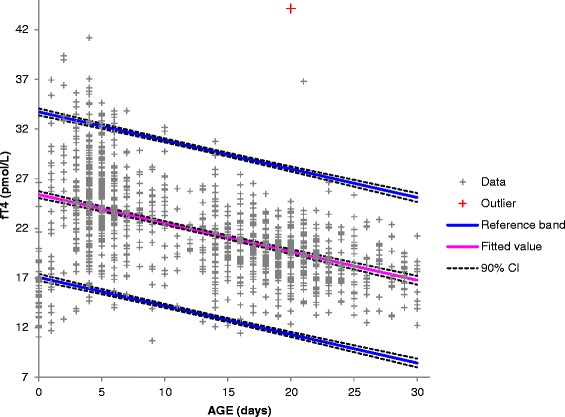
Distribution of fT4 values in relation to age

**Fig. 4:**
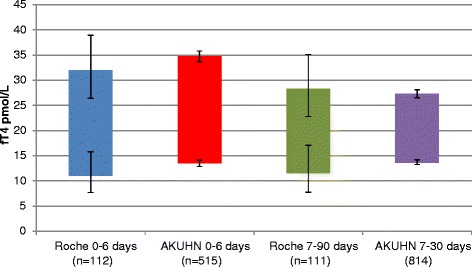
Roche and AKUHN neonatal free thyroxine (fT4) reference intervals with 90 % confidence limits

There was a decline in fT4 levels with increase in age as shown in Fig. 3. The upper and lower limits of the derived RI for neonates less than 7 days old were higher than the ones proposed by Roche. For neonates aged 7–30 days, the derived RI had a higher lower limit and a narrower interval compared to the one proposed by Roche as shown in Fig. 4.

There was a statistically significant difference in the fT4 means across the 4 age stratifications. Gender-wise comparisons in the different age strata were not statistically significant (*x*^2^(3) = 379.601, *p* = 0.000). RIs were subsequently derived for the different age strata as shown in Table 2.

**Table 2:** AKUHN non-parametrically derived neonatal reference intervals for free thyroxine (fT4)

Age (days)	Number (gender)	Lower Limit (pmol/L)	Median	Upper Limit (pmol/L)
		(90 % confidence limits)		(90 % confidence limits)
0 to 7	259 (M)	13.62 (12.98–14.16)	25.10	34.75 (33.64–35.67)
	293 (F)			
8 to 14	73 (M)	13.45 (10.68–15.44)	22.27	30.17 (27.93–32.18)
	72 (F)			
15 to 22	215 (M)	14.16 (13.26–14.63)	19.56	24.80 (23.98–25.48)
	250 (F)			
23 to 30	87 (M)	13.26 (12.23–13.90)	18.15	23.37 (22.39–26.64)
	80 (F)			

Key: *M* Male, *F* Female

## Discussion

There is scanty data on the prevalence of CH in Africa most likely due to a greater focus on infectious diseases which are major causes of morbidity and mortality in children [23]. It is therefore not surprising that there is scarce data on RIs for neonatal TFTs from the African continent. To the best of our knowledge, this is the first study to publish neonatal RIs for TFTs from Africa.

In order to avoid the lengthy and expensive process of establishing RIs, many clinical laboratories adopt values from in-vitro diagnostic company kit inserts, text books or published literature. This is despite the possibility that the populations used in deriving such RIs may be different from the populations served by the respective laboratories. There are very few published studies on neonatal RIs for TFTs partly due to the difficulty in enrolling neonates. The CALIPER study which was carried out in Canada and has so far enrolled 8500 children from birth to 18 years of age has established RIs for many analytes including TFTs [24]. Carrying out a formal RI study is extremely challenging given the need to standardize all phases of the laboratory testing cycle and the significant cost involved. In the absence of a formal RI study, hospital data especially from a primary care setting can be used as an alternative especially for tests that are performed routinely to screen for conditions whose prevalence is not high [25–27]. Given that the prevalence of CH is between 1 in 3000 to 1 in 10000 new born infants [9, 10, 28], most neonates screened for CH would most likely be healthy and serve as appropriate reference individuals. Zurakowski et al. used hospital data collected between January 1993 and August 1996 to derive RIs for T4, T3, TSH, and fT4. This data was obtained from outpatient records at Children’s Hospital in Boston for patients 1 month through 20 years of age [3]. Kapelari et al. carried out a similar study more recently using hospital data from the Medical University Innsbruck in Austria for children aged 1 day to 18 years of age [4].

In this study, we found 94 % and 98.5 % of TSH values from AKUHN neonates aged 0–6 days and 7–30 days respectively fell within the Roche RIs. For fT4, the percentages were 91.7 % and 99.3 % for the respective age groups. When verifying RIs especially those derived from a population that is dissimilar from your local population, CLSI guidelines recommend that a laboratory can accept them if 90 % of values derived from reference individuals from the population served by the laboratory fall within the RIs being verified [19].

The intent of CH screening programs is biased towards detection of primary CH. Given the log linear relationship of fT4 with TSH, measurement of TSH is preferred as a cost effective approach to screening for primary CH. A slight decline in fT4 results in a significant rise in TSH allowing for the diagnosis of subclinical hypothyroidism and possibly early intervention before development of overt symptoms. There is marked variability in published neonatal TSH RIs most likely due to differences in reference populations arising from genetic or environmental factors. It is, therefore, important to adopt population-specific cut offs for TSH so as to optimize detection of primary CH. Compared to the Roche RIs, both our TSH upper limits for neonates aged 0–6 days and 7–30 days were lower with narrower RIs. Considering that the Roche RIs for TSH were derived from only 223 neonates, our RIs derived from 1639 neonates are more appropriate to the local population served by our laboratory. A standard textbook of clinical chemistry has proposed a TSH RI of 1.0–39 μIU/mL for neonates less than 5 days of age and 1.7–9.1 μIU/mL for those aged 2–20 weeks [5]. These are significantly different from what we have derived and would therefore not be ideal for our population. The TSH RI derived from the CALIPER study for babies aged 4 days to 6 months is 0.73–4.77 μIU/mL. This was obtained from 278 babies with equal numbers of males and females and analysis done on the Abbott Architect i2000 [24]. The low upper limit for TSH published from the CALIPER study most likely is a consequence of including babies as old as 6 months of age in the same group as neonates despite the known decline in TSH and fT4 with increase in age within the first year of life. Kapelari et al. determined a TSH RI for neonates of 0.7–18.1 μIU/mL and demonstrated a marked decline in TSH values in the neonatal period very similar to what we have observed [4]. Unexpectedly, we found a significant difference in the distribution and mean rank values between male and female neonates aged 14 days and below. The female neonates had a lower RI which is in keeping with a trend observed by Zurakowski et al. for babies aged 1 year and above [3]. Kapelari et al. found that males had higher mean fT3 concentrations but no sex-differences were found for TSH and fT4 between age-matched serum samples. The observation of a significant gender wise difference in mean rank values for TSH is therefore unique and needs to be further investigated as an obvious explanation is not forthcoming.

There was a general decline in fT4 values with increase in age within the neonatal period which is in keeping with what has been published previously [1, 2, 4, 24]. However, compared to the RIs provided by Roche, our fT4 upper and lower limits for the age group 0–6 days are higher and more precise. For the age group 7–30 days, the lower limit is higher and upper limit lower giving a narrower interval than what is provided by Roche [20]. As mentioned earlier, the Roche study that determined fT4 RIs only included 223 babies compared to 1329 in our study. Burtis et al. has published fT4 RIs for neonates aged 1–4 days as 28.4–68.4 pmol/L and 10.3–25.8 pmol/L for those aged more than 2 weeks [5]. For neonates less than 7 days of age, the fT4 lower limit was 13.62 pmol/L. This is significantly lower than what is proposed by Burtis et al. and potentially would reduce the number of neonates that would require unnecessary follow up. In the CALIPER study, the fT4 RI for both male and female neonates aged 5–14 days was 13.47–41.32 pmol/L and 8.71–32.53 pmol/L for those aged 15–29 days. The CALIPER study used values from 264 neonates with equal numbers of males and females [24]. In our study, the lower limit for fT4 for neonates aged 8–14 days is 13.45 pmol/L which is very similar to the CALIPER study. For neonates aged 15–22 days and 23–30 days, the lower limits of 14.16 pmol/L and 13.26 pmol/L respectively are higher than that derived for the CALIPER study. We found no significant difference in RIs between male and female neonates across all age stratifications. Zurakowski et al. found a significant difference between males and females in T4 but not fT4 levels though their study did not include neonates [3]. Djemli et al. found a significant difference in fT4 levels between males and females in the age group 15–17 years [1].

We do recommend the adoption of our derived RIs by our laboratory and anticipate an increase in the number of neonates found to have elevated TSH given the lower upper limits. The age and gender wise stratification of RIs will ensure that the interpretation of fT4 and TSH is based on appropriate cut-offs that are sensitive to the dynamic nature of TFTs within the neonatal period and hopefully this will result in higher sensitivity for detection of CH.

Our study is limited by the fact that we used hospital data without having well defined exclusion criteria to ensure that sick neonates or those with thyroid disorders were not included. However, most of our data is from neonates attending the wellness clinic which largely comprises healthy neonates. We also reviewed medical records for neonates with out-of-range values and excluded those with acute illnesses. None of the neonates had a confirmed diagnosis of CH at the time of carrying out the study though the follow up data was limited to a maximum of 3 years as the review of medical records was done in 2014. We also carried out parametric analysis to determine RIs after excluding outliers but found no significant change. We therefore believe the non-parametrically derived RIs are appropriate for our neonatal population.

## Conclusion

This is the first study from Africa that has published TSH and fT4 RIs for neonates and will go a long way in providing a guide for the interpretation of TFTs especially when setting up a CH screening programme where the target population is largely a black African population. The differences seen when compared to other published RIs may be reflective of a difference in reference populations or analytical methodologies. More studies from a similar population across the African continent will help verify the findings of this study.

## Abbreviations

AKUHN: Aga Khan University Hospital Nairobi
ANOVA: Analysis of variance
CH: Congenital hypothyridism
CLSI: Clinical Laboratory Standards Institute
fT3: free triiodothyronine
fT4: free thyroxine
IFCC: International Federation of Clinical Chemistry
IQR: Inter Quartile Range
RI: Reference interval
SPSS: Statistical Package for the Social Sciences
T4: Thyroxine
TFT: Thyroid function test
TSH: Thyroid stimulating hormone
