# Erratum to: Reference intervals for thyroid stimulating hormone and free thyroxine derived from neonates undergoing routine screening for congenital hypothyroidism at a university teaching hospital in Nairobi, Kenya: a cross sectional study

**DOI:** 10.1186/s12902-017-0171-9

**Published:** 2017-03-29

**Authors:** Geoffrey Omuse, Ali Kassim, Francis Kiigu, Syeda Ra’ana Hussain, Mary Limbe

**Affiliations:** 1 Department of Pathology, Aga Khan University Hospital, P.O. Box 30270–00100, Nairobi, Kenya; 2 Department of Paediatrics, Aga Khan University Hospital, P.O. Box 30270–00100, Nairobi, Kenya

## Erratum

After the publication of this article [1], one of the authors noticed that the wrong image had been used for Fig. 1 (a duplicate of Fig. 3). The correct Fig. 1 is shown below. This mistake was carried forward by production, and thus, is not the fault of any of the authors

**Fig. 1:**
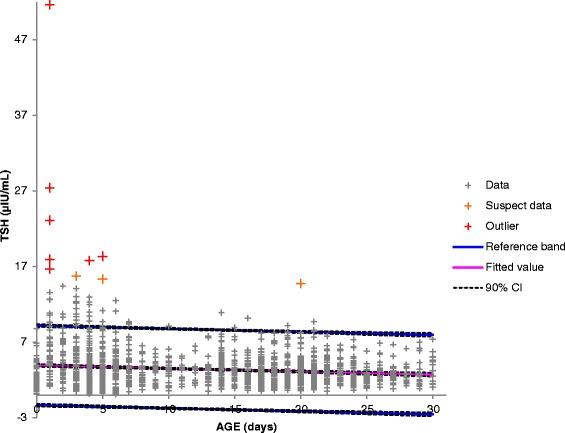
Distribution of TSH values in relation to age
